# Supplementary material for: Mixed-methods exploration of the knowledge of young adults about blood donation processes; a one-center cross-sectional study in a tertiary institution
Source: PLoS One. 2024 Jan 5;19(1):e0295600. doi: 10.1371/journal.pone.0295600 (PMC10769017; doi:10.1371/journal.pone.0295600)
Supplement: S1 File — (DOCX) [file pone.0295600.s001.docx]

**FOCUS GROUP DISCUSION (FGD) 1**

**FGD with non-donor participants**

**Introduction**

The moderator welcomed all participants and introduced herself. She explained that the discussion was for academic purposes only and will be recorded. Some ground rules were set which included that there was no wrong answer, and phones should be put on silent to prevent distractions. Members were assured to feel free and comfortable and pick the refreshments when the need arises. and that the session is being recorded.

**Moderator:** To begin with, we made you aware of the topic from the questionnaire, which is exploring the challenges of blood donation among the students of the University of Cape Coast. We want to get more knowledge about it because filling in yes and no, I agree won't give one the leverage to talk about your feelings towards it but meeting you one on one will be an opportunity for us to get to know more about what is ongoing as far as the donation is concerned. The world health organization (WHO) has made it a point that if about 1-3% of a country's population is to donate blood, we wouldn't run into a deficit. So, when the need arises for blood in the hospital, it wouldn't be that we would have to call for somebody to donate blood before we get blood for the patient, that is what the WHO has stipulated but this is not what is going on in our various hospital facilities. So, we want to find out since UCC is a youthful population (the youthful population are the people who are to be involved as far as the donation is concerned) so we want to find out what is going on, what are the challenges that are making a lot of people not donate blood in our hospitals. To start, we'll like to know our names, so I'll be your moderator, I'm Lizzy as I said earlier on. So, we have Alhassan, Justine, Godfred and Belinda, who'll be taking notes. So, I've mentioned my colleagues’ names. So, when you mention your name tell us the department you're coming from. We're from the department of Medical Laboratory Science. We will start with our brother,

**Responses:** Participant 1: I am from the department of History; Participant 2: I am from the department of B. com and supply chain management; Participant 3 from the history department; Participant 4 from the medical laboratory science; Participant 5 from the department of biochemistry; Participant 6 from the Department of Statistics; Participant 7 from department of Imaging.

**Moderator: So, you're all welcome. First of all, when we say blood donation what comes to mind? Don't forget there's no wrong answer. Just feel free and let's have a nice discussion. It's a maximum of 1 hour.**

**Responses:** “Just donating blood”, “Giving out blood to those who need it”, and “Voluntary action of giving out blood to those who need it.”

**Moderator: Do you think blood donation is a necessary procedure that should be undertaken in our hospital facilities?** Give us a thumbs up if yes, thumbs down if no, and a blow if neutral. So, we're getting 3 people giving thumps up, no thumb down, and the neutral is 2. So those giving thumbs up, why? Participants’ responses were; “Because blood is always needed at the hospitals and at times we'll be running out of blood for and others may need blood but if we take it as a necessary action, it won't be like we're in short of blood and we have to rush to provide some.” “Blood Donation is necessary. It helps us to save lives because sometimes there are accidents and people normally run out of blood and if we voluntarily donate blood, to be stored at the hospital it'll help save lives.” “It helps save lives.” “It's more or less like a normal person doesn't have surgeries at the hospitals and where there's an emergency there's surgery and normally, they get low blood levels, it's best they're given blood, definitely if we donate, so they will get blood because we don't manufacture blood definitely, we get it from other human beings so I'll be best we set aside blood for those who'll need it.” “I think blood donation is a matter of choice and it depends on the situation and the kind of environment that you find yourself despite that not all of us are equal in terms of strength and motivation and there'll always be as you said you maybe a percentage of the population can do that but it doesn't necessarily mean that all of us have to do it.” “I will also say that it's not necessary for you to be giving blood to the people because at times at times we'll have different blood types so if we always go about giving blood each day and all and anytime, I don't think it's necessary.”

**Moderator**: Our sister is saying all of us have different blood types and so we don't have to go about donating, does it mean we all agree with that? Any idea whether we have different blood types and hence shouldn't be donating?

**Responses:** “We know we have different blood groups but as we're sitting here, we’ll get two or more people having the same blood type, and then normally people always get sick and short blood, if you donate your blood and it's O and someone will come to the hospital and need that same blood type so irrespective of the blood type you can still donate.” “I'm not a science student but I think I have some knowledge that some people are universal donors and others are universal recipients so I don't know whether some people can't give blood per their group but if there's a blood group like that, then I think it wouldn't be necessary based on what our sister is saying.”

**Moderator**: Does anyone have an idea since he's saying until your blood matches with someone you're not supposed to donate?

**Responses:** We have different blood but some are compatible, and some are not. To clarify what I said, I might be A and you might be A+ so you have other people who are universal donors, who I think are AB depending on whether you're positive or negative which also counts. So, depending on your blood group and mine, if I donate to you and your antibodies does not fight against those blood group, it wouldn't affect you. Before you'll be given a particular blood, all those things are noted down, so let's say you're B, if B is allowed for you, it will be given to you so it'll be stored.” “It's necessary for us to donate because when you donate, let's say you have a different blood group to mine so I might not need it but someone will need it, so let's say we're in UCC and we all go to UCC hospital to donate blood and we are all B positive, and our moderator comes and does not need that blood, but someone else might come from a different place who needs that B positive blood we've all donated and blood can be stored for some time or even people from other hospitals can come for such blood if there's the need for it.”

**Moderator**: So, our brother is talking about emergencies and exchanging blood as far as the facilities are concerned. **We'll proceed by asking, since we're a youthful population it looks like, in all the questionnaires we circulated, all of us stated that we've never donated before, what is preventing us from donating? Is it that we have some fears in us or some issues that are why we're not donating?**

**Responses;** “For me, the practitioners who'll be undertaking the procedure, if I don't see your certificate that you're qualified enough, I'll not come. What I have seen is that you'll go to the hospital and they'll let someone do it, they'll put it in and pull it out, and you find some others having challenges. “

**Moderator:** **Please who sides with our brother on what he said? That before you put a needle in my skin, I have to see your certificate before I give you the chance?**

**Responses;** “What he's saying is very important because, at the end of the day, it's your body that they'll be putting something in so if they don't put it at the right place, you might get an infection or a different (remaining audio not heard).” “Not necessarily a certificate or something, but you should be able to know that this person can do the job. Seems one-sided, you have to lol at the person before you allow it because we can't sit down for the person to be piercing your body and we feel pain so we wouldn't want that.” “So, I agree with what my brother said. In our settings, I won't say that the doctors are lazy but I don't know whether they like delegating so much. They just leave the thing to the person to do the job and then for me, my body is small. The other time I went and they gave me to an intern and it didn't end well and when the person with the qualification came, it was easier.” “Sometimes you go to the hospital and they give you to those who are coming to learn and they're not qualified and they end up doing it anyhow and I'm not ready for such a thing to happen to me.”

**Moderator:** Since we're done with his point, we'll go on, **what at all is preventing us from donating?**

**Responses;** “As for me, I'm afraid of catching diseases. The equipment they use is old.”

**Moderator:** Please how many of us are facing the same at the facilities we use that their equipment is archaic?

**Response**: For the facility I use, anything that they use in blood donation, it has to be a new one be it the blood bag or needle”, “It's always new so for that one, it's not preventing me.”

**Moderator:** Please what again?

Response: “Sometimes, some background knowledge before donating is necessary, for me like this, I'm not a science student so for some of the things I'm not able to verify them so once a time, a person told me that if you donate your blood, you have to subsequently donate and I don't know how I'll be able to do that.”

**Moderator:** Please who has encountered that situation before?

**Response:** “And also my brother once told me that when you donate blood, you become weak.” “And also, I think it's a lack of knowledge on donation. For instance, I don't know much about blood donation. I've heard it's good and not good and that stuff so the education as to whether it's okay or not, I don't know.” “I think what is preventing me is, **I remember they once came to our community for blood donation, I went and they performed a test and from the test, they just told me I don't qualify. And the next time at a different place too, they just told me I don't qualify to donate and that was it. I was not told what it was that did not qualify me. It was until recently when I was at the lab that was performing FBC that I was told that my blood level was low. So, it was then that I reflected that it may be because of this that is why I was told that I couldn't donate.** So, if I was told by those people that it was because of my low blood levels, I'll work on it or they gave me advice on how to improve my blood donation, so next time I'll go and donate.” “One thing I've noticed is, I have no interest in donating.”

**Moderator**: We've raised concerns, but **what do we think could be done to remedy the situation?**

**Responses:** Sometimes we feel like in this country, we know that blood donation is done willingly but I've heard that for some donations, NGOs do make about it that after the person has donated blood, give it to them but you find that most of the hospitals especially the public hospitals don't give it to the donors but if the comparison is made to some countries like the U.S.A., they buy it and so at least they can give the donors incentives to motivate them.” “With the challenges too, I also know that some of them sell blood to the donors, we know it saves lives but they're selling it to the person it's supposed to save the life so why will I go and donate if they're going to sell it to the patient.” “We have to go in for qualified people to do the job.” “Motivation for donors.”

**Moderator:** **What can be done to help the facilities in terms of qualification?**

**Responses: “**Health workers should be taken through workshops to develop them and they shouldn't be where they are but they should explore and make comparisons with other Europe countries who are good in this area. “Talking about the qualifications, when we enter the facilities, we always have in mind that some of them are qualified, but as we say that we go and they'll be pinching you but if we have a particular place that we have everything that shows that this is where blood is taken, so when we come, we know that those there are qualified.” “Sometimes when we go to the hospital, we know that the lab is for donating. It'll be best if they're being trained with inanimate objects first on how to take place blood samples instead of using humans as objects for practice.” “There should be adequate information on donation to remove the misconceptions that people have, and to be able to know the misconceptions, I think we should do research like you're doing and be able to talk them out of it with advice to donate. It can be done using multimedia.” “Education is very important. I remember one of our supervisors said that you're not paying for the blood but rather the blood bag and stuff. And sometimes people don't come to donate blood but they find boys to come and donate and assure to give them money. So, because the person will be given the money, he'll come and donate, and when the person pays for it, if a recipient comes for it, they'll also be charged. But if we're all educated to donate and we're all donating, definitely there shouldn't be any charges and if we're to pay for it, it should be just for the price of the bag which is at a lesser price.” “I think we should have some correlation with the price.” “I will say the information, so let's say in the University like those that they're in the health sector that knows about blood groups can go should have some particular days that they go the halls to educate people about it and give them knowledge about what goes into donating blood, what you should do and not do and people will get the knowledge and start coming to donate.” “Still, on the information, there are some people that are easily influenced when you talk to them about something. We can target schools, mosques, and churches. If we point out these things at such places, people will be influenced to donate.” “We can also use influential people who are knowledgeable in blood donation to educate people on it.” “I think there should be control of the blood we take and account for it. These were the number of blood donors who gave blood and this was how we used the blood. I think if there's that transparency, even if it costs, it should be something minimum and this can motivate donors to also donate.” “From my experience, I think if there was something like counseling after the practitioner says you are not eligible to donate. I could have worked on it to donate the next time. And secondly on the issue of education, just as my colleagues said that we reach out to the communities because I remember that it was after the Friday prayers that they come to inform the members that maybe next week Friday we'll be coming for blood donation. That is where I normally get to have that information and on all those two experiences, I made mention of it being in the mosque but I feel like there should be some connection with the people. Let's say it's Tafo hospital and you want to have people donating blood, you should also have something to be given to the community so let's say once in a while you go to the community and educate them on let's say disease A or B free and let the people come out with their questions. There will be that friendship between the hospital and the community so they feel at ease to donate when you tell them to do so. Today you come and educate them and you tell them there'll be a donation next week/month then there will be that vibe so whenever you want something from them, it'll be easy to get it from them. Rather you sit there, when you feel you have a shortage of blood then you come there.” “I also think you should let the donors be aware of the number of bags of blood they'll take from that person. A friend of mine donated when we were in school and he complained that they took all his blood.”

**Assistant Moderator**: How d**o you think religious reasons can serve as a hindrance to someone donating?**

**Response:** Yeah, I have a friend who is a Jehovah’s witness, he said it wasn't good to donate and backed it with scriptures. They believe that blood is life hence when given to someone can transmit bad traits to another person and end up taking one's lifestyle”.

**Assistant moderator:** **What are the important things that we have to take notice of in this discussion?**

**Response:** “I think it has to do with the information/education with knowledge”

**Assistant moderator**: Do you think that aside from the interns, the actual workers are not certified enough?

**Responses:** “Partly, because if they're that certified it would be incumbent on them to also train the interns well.” “I think those working there know how to do it because they can do it when the interns are struggling.”

**Conclusion**

A summary of the discussion report was read by the assistant moderator and agreed upon by the participants at the end of the discussion. It captured that blood donation saves lives, but most challenges centered on the fact that most people had misconceptions as a result of lack of education. The remedy suggested was there should be more education on blood donation and transparency from the various stakeholders and establish rapport with the various communities before blood is needed.

**FOCUS GROUP DISCUSION (FGD) 2**

**FGD with participants with prior blood donation experience**

**Introduction**

The moderator welcomed all participants and introduced herself. She explained that the discussion was for academic purposes only and will be recorded. Some ground rules were set which included that there was no wrong answer, and phones should be put on silent to prevent distractions. Members were assured to feel free and comfortable and pick the refreshments when the need arises. and that the session is being recorded.

**Moderator:** To begin with, we made you aware of the topic from the questionnaire, which is exploring the challenges of blood donation among the students of the University of Cape Coast. We want to get more knowledge about it because filling in yes and no, I agree won't give one the leverage to talk about your feelings towards it but meeting you one on one will be an opportunity for us to get to know more about what is ongoing as far as the donation is concerned. The world health organization (WHO) has made it a point that if about 1-3% of a country's population is to donate blood, we wouldn't run into a deficit. So, when the need arises for blood in the hospital, it wouldn't be that we would have to call for somebody to donate blood before we get blood for the patient, that is what the WHO has stipulated but this is not what is going on in our various hospital facilities. We want to find out since UCC is a youthful population (the youthful population is the people who are to be involved as far as the donation is concerned) what is preventing us from regularly engaging in blood donation activities since we have all donated before? Before we proceed, we'll like to know our names, so I'll be your moderator, I'm Belinda as I said earlier on. We have Alhassan, and Godfred who'll be taking notes for clarification and transcribing. So, I've mentioned my colleagues’ names. So, when you mention your name tell us the department you're coming from. We're from the department of Medical Laboratory Science.

**Responses:** Participant 1: I am from the department of Nursing; Participant 2 from the statistics department; Participant 3 from Early childhood department; Participant 4 from the department of Medical Laboratory Science; Participant 5 from the Department of B.com accounting; Participant 6 from psychology and anthropology department; Participant 7 from the Department of Medical Laboratory Science; Participant 8 (department not heard in audio).

**Moderator:** I welcome you all once again. To begin with, **do we all think that blood donation is a necessary procedure? And why?**

**Responses:** “You have to understand whom you're donating to that's a fellow human being. Regardless of my religious or moral principles, I think it's a humane principle that I give blood to my fellow human beings who'll need blood.” “Blood is meant to save a life, I'll donate blood to whom I know, my close relative because there are instances that you'll donate blood but when you need blood, they'll tell you that there's no blood. There was this incident that happened this year, my wife was in labor and needed blood urgently though knowing her blood group and mine were the same. So the midwives told me to go for blood from the blood bank so I could replace it later. I was there for 2hrs and they told me there was no blood until they took it from me. Surprisingly, when they opened their refrigerator, I saw different types of blood so I asked myself why there was blood but they're failing to give it to me. That is why I agree that it saves life because through that I was able to save my wife and baby.” “My reason is that you donate to save lives.” “I'll donate blood to someone I know, blood is in components, so if someone needs blood, which part is the person in need of? I'd prefer to donate the specific component the person needs instead of the whole blood.”

**Moderator: What were the fears we had before we donated blood?**

**Responses:** “I was told when you donate blood, you lose a lot of blood and might not recover it as quickly as possible. I'll be feeling dizzy and might lose a lot of blood.” “I know that sector. I used to have blood in excess above the normal range so I used to donate it so I wasn't afraid of anything when it was time to donate it.” “A friend called me that his sister needed blood, my fear was before the blood is taken, they would have to test and I was afraid of the results because I didn't know my state.” “The reason why I was not afraid was because of the experience I had at a young age where a friend had an accident and bled profusely of which a donation from the older brother saved him. Ever since then, I see donation as a chance to save a life.” “I had to be bold in other to donate blood for my brother.” “It was a mixed feeling for me, the blood bank came to our chaplaincy and we donated for them.” “I knew at the time that my wife was going to deliver, that they would request for blood so I psyched my mind and removed any fear factor.”

**Moderator: After the donation, were your fears confirmed?**

**Responses: “**When I finished, I realized it was simple and not like what I heard earlier about blood donation. The procedure was so smooth, it was just my chronic eye problem that I experienced.” “When I was recruited to donate, the professional wasn't friendly a bit. Bring your hand, and took a big needle so my heart was beating fast and that made me regret my decision to donate before and even afterward.” “At least there should be some kind of consolation from the staff and they should explain the procedure during the process.” “I was lured to donate so I was pampered due to that young age and so I didn't experience that. So, they were friendly.” “There should be some kind of pampering to take away the anxiety the donor might be feeling.” “In all everything was good right from the reception where I did my donation.”

**Moderator: When allowed to donate, will we?**

**Responses: “**I will donate to my relatives but not the national blood bank because you'll need blood when it's your turn and they'll tell you there's none.” “I will do it again because they gave us a card as a receipt to send when the need for blood arises. It served as a motivation for me to donate again.” “I will donate again because it saves lives.”

**Moderator:** **Will you donate blood to someone you don't know?**

**Responses: “**I will not do that because you'll need blood when it's your turn and they'll tell you there’s no blood. At least if I ever need blood or my relative is sick and needs blood there should be some compensation.” “My experience was so bad that after donating, I lost a lot of weight. I didn't know so much and the education they gave me was less. I may but I'm not sure I'll convince someone to do so if the person wants to do it then fine.”

**Moderator: Do you think that there are challenges in the system that needs to be addressed?**

**Responses: “**One is inadequate education given to the one donating the blood and the time spent in donating blood is another factor. Another one is inadequate privacy and facilities.” “There should be a demarcation from where we do the consultation to where we donate the blood.” “Inadequate data for blood donated at the blood bank. The amount of blood that was donated and the recipients. Every year they come for blood but they don't give account.” “There should be transparency as well.” “Lack of motivation for donors.” “There should be well-trained personnel and good communication from them.” “Screening before the actual donation. The tests done can be modified other than HIV, VDRL, Hepatitis B, and C. There should be an additional test to ensure the safety of the blood by using molecular techniques before being transfused to the patients.” “Another challenge is the labeling of the samples which is sometimes done wrongly.” “Poor storage facilities. The refrigerators have different temperatures from what the thermometer is reading. There were instances at the hospital where part of the blood sample in the refrigerator was frozen so how will the blood be defrosted when a patient needs it urgently?” “The education on blood donation should be intense.” “I wish in future we'll have properly trained personnel who'll solely handle blood donation issues. After my donation, I inquired about the guy who took my blood and got to know that he was someone who had been trained from the bench. He has just 6 months of training whiles someone had spent 6 years doing the same thing. So, you can imagine the kind of services such a practitioner would give.” “Another challenge is inadequate staff because in some places one laboratory technician handles all things related to the lab. They should be more so each person does a specific task and some of the challenges can be curtailed.” “Assuming you're the head of lab or personnel, what can you do to help eliminate these challenges?” “They should be regular in-service training for personnel across the country.” “Training the personnel on technology advanced too is necessary and the youth in the senior high schools should also be educated on blood donation.” “The in charge should be open to the staff to address their challenges.” “Some facilities do not also have incinerators.” “If there's an opportunity for any of the personnel to undertake a short course to help the facilities, they should be allowed to do that.” “They should provide modern facilities like refrigerators.” “Motivation for donors, not just in food items but cards.” “Adequate preparation of the donor.” “What will you do as the minister of health to encourage the youth to engage in donation?” “A module can be incorporated into the syllabus of students to educate students on it.” “A liaise with the national agency of communication to help sensitize the Civic society on the pros and cons of donation.” “Students at the senior high school should be fed well also and also build ultramodern medical laboratory school so that their training will be intensified.”

**Conclusion**

The discussion report was read by the assistant moderator and agreed upon by the participants.
