# Supplementary material for: Mixed-methods exploration of the knowledge of young adults about blood donation processes; a one-center cross-sectional study in a tertiary institution
Source: PLoS One. 2024 Jan 5;19(1):e0295600. doi: 10.1371/journal.pone.0295600 (PMC10769017; doi:10.1371/journal.pone.0295600)
Supplement: Attachment — Submitted filename: Authors Point-by-point responses to reviewer report.docx [file pone.0295600.s003.docx]

**Point-by-point responses to reviewer comments (manuscript ID: PONE-D-23-21319)**

Title: Mixed-methods exploration of the knowledge of young adults about blood donation processes; a one-center cross-sectional study in a tertiary institution.

| **Comment** | **Response** | **Page** |
| --- | --- | --- |
| Academic Editor |  |  |
| 1. Please ensure that your manuscript meets PLOS ONE's style requirements, including those for file naming. | Noted.  The manuscript has been revised to meet the PLOS ONE style requirements. |  |
| 2. Please provide additional details regarding participant consent. In the ethics statement in the Methods and online submission information, please ensure that you have specified (1) whether consent was informed and (2) what type you obtained (for instance, written or verbal, and if verbal, how it was documented and witnessed). If your study included minors, state whether you obtained consent from parents or guardians. If the need for consent was waived by the ethics committee, please include this information.  If you are reporting a retrospective study of medical records or archived samples, please ensure that you have discussed whether all data were fully anonymized before you accessed them and/or whether the IRB or ethics committee waived the requirement for informed consent. If patients provided informed written consent to have data from their medical records used in research, please include this information. | Under the materials and methods section, a section on ethics has been include:  **Statement of Ethics and Human Subjects Issues**  Ethical approval was obtained from the University of Cape Coast Institutional Review Board (ethical clearance ID: UCCIRB/CHAS/2022/77). Our study protocol conformed to the provisions of the Declaration of Helsinki in 1995 (as revised in Brazil 2013) including confidentiality, risks and benefits assessments, consent to participate, and ensuring respect to participants. It was ensured that written informed consent was sought from participants and that the information given was kept confidential. Informed consent was acquired by explaining the research to the participants to understand before engaging them; since the age of consent is 18 years in Ghana, introductory letters were sent to obtain accent from the parents of participants who were less than 18 years old. It was also ensured that participants were not coerced into participating but joined voluntarily and that the evaluation did not cause any harm to the participants. The anonymity of participants was ensured by not recording information in a way that links participants’ responses with identifying information. Moreover, participants had the right to withdraw their consent and discontinue participation from the study at any time. The information obtained from the research was used for the intended purpose only. Although participants who consented to participating in the FGD were initially required to provide phone numbers, these were removed from the data after the FGDs to maintain data anonymity.  Not applicable | Page 10, line 177 - 193 |
| 3. Your ethics statement should only appear in the Methods section of your manuscript. If your ethics statement is written in any section besides the Methods, please move it to the Methods section and delete it from any other section. Please ensure that your ethics statement is included in your manuscript, as the ethics statement entered into the online submission form will not be published alongside your manuscript. | Noted. The ethics statement has been moved to the materials and methods section. | Page 10, line 177 - 193 |
| 4. Please include captions for your Supporting Information files at the end of your manuscript, and update any in-text citations to match accordingly. Please see our Supporting Information guidelines for more information | Noted.  The Supporting information files have been moved to the end of the manuscript.  In-text citations are in sync with the respective names assigned to the supporting files. | Page 7; line 123 – 124  &  Page 9, line 150 - 151 |
| 5. Please review your reference list to ensure that it is complete and correct. If you have cited papers that have been retracted, please include the rationale for doing so in the manuscript text, or remove these references and replace them with relevant current references. Any changes to the reference list should be mentioned in the rebuttal letter that accompanies your revised manuscript. If you need to cite a retracted article, indicate the article’s retracted status in the References list and also include a citation and full reference for the retraction notice. | Reference list has been checked for completeness. |  |
|  |  |  |
| **Reviewer 1** |  |  |
| Despite the fact that it is a one-center study with a limited sample size, the manuscript is technically and grammatically good. | Noted. |  |
| But I'm not convinced by the study's rationale. Meeting the annual blood requirement of a country directed donation is also crucial, as is replacement donation. Voluntary blood donation requires 'Education'; in other circumstances, 'Motivation' is required. Unfortunately, this study is just concerned with "Education". | While we agree with the reviewer that both directed donations and replacement donations are important, in view of the fact that these two modes of blood donations are associated with higher incidence of TTIs, the WHO has been advocating for 100% voluntary donations which has been noted to be associated with lower incidence of TTIs (WHO-IFRC. Towards 100% Voluntary Blood Donation: A Global Framework for Action. Geneva: World Health Organization; 2010. p. 138) https://www.who.int/publications/i/item/9789241599696.  Our rationale for the study was informed by the hypothesis that educational attainment could be an important factor when considering measures to improve blood donation. This informed the choice of tertiary students as sampling frame for the study. Our overarching aim therefore, was to quantify the knowledge of tertiary students regarding blood donation, and identify the perceived challenges that these youth face regarding voluntary blood donation as well as identify actionable points that could be harnessed to improve blood donations rate per 1000 population to improve access to blood products across the country.  The **background** has been revised to include additional information “Paradoxically, the developing countries however have a majority youthful population compared to the ageing population in the developed countries. All things being equal, this overly youthful population should potentially translate into adequacy of donor blood stocks. Presently, the Ghana healthcare system is only able to approximately meet 60% of its annual blood transfusion needs [1]. Previous studies in the SSA region have found altruism [2, 3], a sense of communal collectivistic outlook [4, 5] and reciprocity of blood donation [6, 7] as factors that could be harnessed to motivate the populace to increase blood donation rate per a given population.  However, a previous cross-sectional study among the general populace in the northern region of Ghana found that approximately one-third of blood donors knew the minimum age for blood donation or blood donation frequency per year [8] suggesting poor knowledge in blood donation-specific processes. Other studies elsewhere have also similarly indicated that education might be an important consideration in the drive towards achieving donor blood sufficiency [9-12]. Tertiary students within the SSA context, by virtue of national representation as well as meeting donor age eligibility remains a potential resource in efforts to maximize blood donations across the country [13]. When sufficiently motivated, these tertiary students could be permanent voluntary, non-remunerated blood donors as well as potentially serve as champions/ambassadors of blood donor mobilization drives within their respective communities. Hypothesizing that the endemic apathy towards blood donations could be a function of educational attainment, we sought to explore the perspectives of young adults in a tertiary educational setting to assess their knowledge regarding the blood donation requirements and procedures.” | Pages 3 & 4, line 45 - 65 |
| I have no idea about statistical analysis | Noted |  |
|  |  |  |
| **Reviewer 2** |  |  |
| Blood donation is a very important issue, but in addition to the usual need to receive blood, malaria has added an additional burden in Africa. Considering the low amount of public aid in Ghana and the lack of response to the existing needs, the investigation of this issue is of particular importance. | Noted. |  |
| As mentioned in this article, the lack of sufficient information about the accepted criteria for donor selection, the effects of donation on the body's metabolism, and the lack of privacy in blood collection centers are among the things that reduce the enthusiasm of people to donate blood. | Noted. |  |
| Despite revealing valuable findings, this research has not mentioned previous similar research in this field. | Noted.  Relevant studies have been reviewed and included in the background section:  “Previous studies in the SSA region have found altruism [2, 3], a sense of communal outlook [4, 5] and reciprocity of blood donation [6, 7] as factors that could be harnessed to motivate the populace to increase blood donation per 1000 population. However, a previous cross-sectional study among the general populace in the northern region of Ghana found that approximately one-third of blood donors knew the minimum age for blood donation or blood donation frequency per year [8] suggesting poor knowledge in blood donation-specific processes. Other studies elsewhere have also similarly indicated that education might be an important consideration in the drive towards achieving donor blood sufficiency [9-12].. Tertiary students within the SSA context, ...” | Pages 3 – 4, line 45 – 65. |
| Also, the discussion section contains a lot of unnecessary information, including quotes from participants, which could have been removed. To increase the quality of the article, I suggest rewriting the introduction and discussion using more relevant articles. | Our reporting followed the COREQ Guideline for reporting qualitative studies <https://doi.org/10.1093/intqhc/mzm042>  As stated in the domain 3 (analyses and findings), item 29 (reporting) states that participants’ quotations should be presented as elaborated in “Were participant quotations presented to illustrate the themes / findings? Was each quotation identified? e*.g. participant number”*  The exact quotations are thus an important component of qualitative data presentation to capture participants responses. This is what separates qualitative data reporting from quantitative data. The root epistemology that underpins the quantitative paradigms emphasizes the perspective of the disinterested outsider, quantification and standardization of measurement, high levels of controls of threats to validity and the goals of general (if not universal) explanations.  Regarding the introduction, additional relevant articles have been referenced as recommended “Relevant studies have been reviewed and included in the background section:  “Previous studies in the SSA region have found altruism [2, 3], a sense of communal outlook [4, 5] and reciprocity of blood donation [6, 7] as factors that could be harnessed to motivate the populace to increase blood donation per 1000 population. However, a previous cross-sectional study among the general populace in the northern region of Ghana found that approximately one-third of blood donors knew the minimum age for blood donation or blood donation frequency per year [8] suggesting poor knowledge in blood donation-specific processes. Other studies elsewhere have also similarly indicated that education might be an important consideration in the drive towards achieving donor blood sufficiency [9-12]. ” | Pages 3 – 4, line 45 – 65. |
| The text is clearly written but has minor grammatical problems. For example, in the second and subsequent repetitions, use the abbreviated form "SSA" and mention the word "WHO" in full in the first place. | Noted. |  |
|  |  |  |
| **Reviewer 3** |  |  |
| In this paper title ‘Mixed-methods exploration of the knowledge of young adults about blood donation processes; a one-center cross-sectional study in a tertiary institution’: Belinda Baidoo and colleagues evaluate the knowledge of university students about blood donation processes with the aim of exploring the perspectives of young adults in a tertiary educational setting to assess their knowledge regarding the blood donation requirements and procedures. The study included 382 students (18 – 49 years) studying at the University of Cape Coast (UCC) in the central region of Ghana. The students’ knowledge, perspectives and opinions about blood donation processes were assessed using a semi-structured questionnaire and focus group discussion. In their conclusions, the authors highlighted that irrespective of the educational attainment and the passage of time, knowledge about blood donation practices among Ghanaians are not improving and requires urgent attention. This is an interesting study addressing clinically relevant issues of lack of knowledge about blood donation practices among population and the necessity of creating awareness among the community to minimize the global shortage of blood donation. The following few points however deserve attention. | Noted. |  |
| 1. In the Background section (page ‘3’ line 31), sub-Sharan Africa (SSA). Probable a Typo mistake, if so, needs to be corrected. | Sharan has been revised to read “Saharan” | Page 3, line 30. |
| 2. In the material and methods/ Inclusion/exclusion criteria section (page ‘5’ line 78-81), and in the sampling of participants section (page ‘6’ line 97-98). The authors stated that regular undergraduate and post graduate UCC students were included in this study, and that others students groups (sandwich and distance learning students) were excluded. It would be better if the authors could discuss in the manuscript the reason(s) of this inclusion/exclusion criteria. | Noted.  The revised manuscript has acknowledged this as a limitation in the last paragraph which include “We acknowledge that the fact that only one tertiary institution constituted our sampling frame means that our findings may not necessarily be representative of all universities in Ghana. Additionally, in view of the face-to-face focus group discussion that followed the initial questionnaire data collection, sandwich students and distance learning students were excluded from the study; this might have potentially impacted the generalizability of the research outcomes. Moreover, it is also possible that the non-probability sampling technique employed in this study might have potentially led to sampling bias since the data may reflect the experiences and views of those who are more receptive to research.” | Page 26, line 429 – 436. |
| 3. In the material and methods/sample size section (page ‘5’ line 91-92), the authors stated that “a total of 420 questionnaires (10% allowance) were distributed to students” whereas, in the Figure 1 (page ‘7’): The authors indicated that “424 questionnaires were distributed recruited participants”. This discrepancy needs to be rectified. | The manuscript has been revised to read “a total of 424 questionnaires (10% allowance) were distributed to students”.  The estimated sample size of 385 plus estimated 10% (39) allowance equals 424. | Page 6, line 99 |
| 4. In the material and methods/ data collection section (page ‘7’ figure 1 [A schematic of the procedures for the data collection]), in the figure, that total number of questionnaires that were filled/not returned (382 +20 +18) does not add up to the total number of questionnaires distributed (424): this needs to be corrected. | Noted.  The figure has been revised to capture 22 for the non-returned questionnaires. | Page 7 |
| 5. In the results section (page ‘10’ table 1 [The socio-demographics of study participants]), the students age ranged from 15 - 49 years, however, the age of recruited 382 young adults stated in the abstract (page’2’ line 10-11) is 18 – 49 years. This need to be rectified. | The abstract has been revised to correctly read 15 – 49 years. | Page 2, line 10 |
|  |  |  |
|  |  |  |

**References**

1.	WHO. Ghana commemorates World Blood Donor Day; Safe blood for all. Geneva: World Health Organization; 2019.

2.	Asenso-Mensah K, Achina G, Appiah R, Owusu-Ofori S, Allain JP. Can family or replacement blood donors become regular volunteer donors? Transfusion. 2014;54(3 Pt 2):797-804. Epub 2013/04/26. doi: 10.1111/trf.12216. PubMed PMID: 23614450.

3.	Adewuyi J, Olawumi H, editors. Factors in the low recruitment of voluntary blood donors among eligible Nigerians. Vox sanguinis; 2006: Blackwell Publishing 9600 Garsington RD, Oxford OX4 2DQ, OXON, England.

4.	Salaudeen A, Odeh E. Knowledge and behavior towards voluntary blood donation among students of a tertiary institution in Nigeria. Nigerian journal of clinical practice. 2011;14(3):303-7.

5.	Sekoni A, Balogun M, Odukoya O, Inem V, Onigbogi O. Blood donation practices and willingness to donate among residents of an urban slum in Lagos Nigeria. Nigerian Postgraduate Medical Journal. 2014;21(1):21-7.

6.	Muthivhi T, Olmsted M, Park H, Sha M, Raju V, Mokoena T, et al. Motivators and deterrents to blood donation among Black South Africans: a qualitative analysis of focus group data. Transfusion medicine. 2015;25(4):249-58.

7.	Asamoah-Akuoko L, Hassall O, Bates I, Adongo P, Bygbjerg I, Ullum H. Socio-demographic characteristics and attitudinal factors in first-time voluntary and family replacement blood donors in Southern Ghana. Vox Sang Sep. 2016;111(1):120.

8.	Mohammed S, Essel HB. Motivational factors for blood donation, potential barriers, and knowledge about blood donation in first-time and repeat blood donors. BMC Hematology. 2018;18(1):36. doi: 10.1186/s12878-018-0130-3.

9.	Saha S, Chandra B. Influence of Age and Education on Blood Donation: A Qualitative Research. Studies on Ethno-Medicine. 2016;10(4):425-35. doi: 10.1080/09735070.2016.11905515.

10.	Lemmens KPH, Abraham C, Ruiter RAC, Veldhuizen IJT, Dehing CJG, Bos AER, et al. Modelling antecedents of blood donation motivation among non-donors of varying age and education. British Journal of Psychology. 2009;100(1):71-90. doi: <https://doi.org/10.1348/000712608X310237>.

11.	Tscheulin DK, Lindenmeier J. The willingness to donate blood: an empirical analysis of socio-demographic and motivation-related determinants. Health Services Management Research. 2005;18(3):165-74. doi: 10.1258/0951484054572547.

12.	Ugwu NI, Uneke CJ, Ugwu CN, Oti WJO, Agbo UN, Akamike IC. Effect of Blood Donor Educational intervention on the knowledge and Attitude towards Voluntary Blood Donation among Medical Students at a Nigerian University. Niger Med J. 2020;61(3):163-8. Epub 2020/10/27. doi: 10.4103/nmj.NMJ_177_19. PubMed PMID: 33100469; PubMed Central PMCID: PMCPMC7547758.

13.	Salma N. Perceptions of blood donation among the students of Jahangirnagar University, Bangladesh. Asian Journal of Medical and Biological Research. 2021;7(4):339-47.
