# Supplementary material for: Mixed-methods exploration of the knowledge of young adults about blood donation processes; a one-center cross-sectional study in a tertiary institution
Source: PLoS One. 2024 Jan 5;19(1):e0295600. doi: 10.1371/journal.pone.0295600 (PMC10769017; doi:10.1371/journal.pone.0295600)
Supplement: Attachment — Submitted filename: Point by point responses to reviewers comments.docx [file pone.0295600.s004.docx]

**POINT-BY-POINT RESPONSES TO EDITOR’S COMMENT (PONE-D-23-21319R1)**

| **Comment** | **Response** | **Page** |
| --- | --- | --- |
| 1. Abstract: Please rewrite the abstract in an unstructured form. | Abstract has been written in an unstructured form | Page 2. |
| 2. Lines 7,8: Revise as follows: (semi-structured questionnaire and focus group discussion [FGD]) | Done. | Page 2. |
| 3. Line 53: Please change northern to Northern | Done. | Line 53, page 4. |
| 4. Line 77: revise to Study site and population | Done. | Line 77, page 5. |
| 5. Line 78: Central | Done. | Line 78, page 5. |
| 6. Lines 81-4: Please check, the total student breakdown doesn’t add up to 74,720 | Total number has been revised to read 74,710 in agreement with the composite numbers. The revised manuscript thus read: “The school had a total population of 74,710 of which 18,949 were regular undergraduate students, 1,445 were sandwich undergraduate students, 1,014 regular postgraduate students, 2,773 sandwich postgraduate students, 48,989 distance undergraduate students and 1,540 post graduate distance students.” | Line 81 – 84, page 5. |
| 7. Line 113: Please check, the breakdown for the 424 questionnaires distributed did not add up to the 424. Also check the first box, there is some typos. Additionally, un-highlight the 22 | Noted.  The numbers in the figure have been revised to correctly read “382 completely filled questionnaires, + 24 questionnaires not returned + 18 questionnaires not completely filled.”  The highlight text has been removed. | Line 111 -112, page 7. |
| 8. Line 132/3: change … ‘since all the participants were tertiary students’ to … ‘since all the participants understand and speak the English language fluently’ | Done.  The revised manuscript thus read “Each FGD session lasted about 60 minutes and was conducted in English language since all the participants understand and speak English language fluently” | Line 132 – 133 page 8. |
| 9. Line 374: Indicate the full form of FBC, on first mention | Done.  The FBC has been defined as “full blood count” | Line 374 – 375, page 23. |
